# Hemispheric differences in altered reactivity of brain oscillations at rest after posterior lesions

**DOI:** 10.1007/s00429-021-02279-8

**Published:** 2021-04-24

**Authors:** Jessica Gallina, Mattia Pietrelli, Marco Zanon, Caterina Bertini

**Affiliations:** 1 Centre for Studies and Research in Cognitive Neuroscience, University of Bologna, Via Rasi e Spinelli 176, 47521 Cesena, Italy; 2 Department of Psychology, University of Bologna, Viale Berti Pichat 5, 40121 Bologna, Italy; 3 Department of Psychiatry, University of Wisconsin–Madison, 6001 Research Park Blvd, Madison, WI 53719 USA; 4 Neuroscience Area, International School for Advanced Studies (SISSA), Via Bonomea, 265, 34136 Trieste, Italy

**Keywords:** Hemispheric differences, Alpha oscillations, Theta oscillations, Resting state, Hemianopia

## Abstract

A variety of evidence supports the dominance of the right hemisphere in perceptual and visuo-spatial processing. Although growing evidence shows a strong link between alpha oscillations and the functionality of the visual system, asymmetries in alpha oscillatory patterns still need to be investigated. Converging findings indicate that the typical alpha desynchronization occurring in the transition from the eyes-closed to the eyes-open resting state might represent an index of reactivity of the visual system. Thus, investigating hemispheric asymmetries in EEG reactivity at the opening of the eyes in brain-lesioned patients may shed light on the contribution of specific cortical sites and each hemisphere in regulating the oscillatory patterns reflecting the functionality of the visual system. To this aim, EEG signal was recorded during eyes-closed and eyes-open resting state in hemianopic patients with posterior left or right lesions, patients without hemianopia with anterior lesions and healthy controls. Hemianopics with both left and right posterior lesions showed a reduced alpha reactivity at the opening of the eyes, suggesting that posterior cortices have a pivotal role in the functionality of alpha oscillations. However, right-lesioned hemianopics showed a greater dysfunction, demonstrated by a reactivity reduction more distributed over the scalp, compared to left-lesioned hemianopics. Moreover, they also revealed impaired reactivity in the theta range. This favors the hypothesis of a specialized role of the right hemisphere in orchestrating oscillatory patterns, both coordinating widespread alpha oscillatory activity and organizing focal processing in the theta range, to support visual processing at the opening of the eyes.

**Supplementary Information:**

The online version contains supplementary material available at 10.1007/s00429-021-02279-8.

## Introduction

There is a longstanding agreement about the existence of hemispheric asymmetries in visuo-spatial abilities (Duecker and Sack 2015; Heilman and Van Den Abell 1980; Kinsbourne 1977). For instance, the right hemisphere seems to have a dominant role in orienting and modulating attentional allocation to both the ipsilateral and the contralateral hemifields, whereas, the left hemisphere is only involved when attending to the contra-lateral hemifield (Heilman and Van Den Abell 1980). Strong evidence about this dominance comes from experimental paradigms in healthy participants using cued shifts of spatial attention (Gitelman et al. 1999; Nobre et al. 1997) and from neuropsychological disorders such as spatial hemineglect, a failure to perceive and respond to stimuli on the contra-lesional side of space, which is more common and severe following right than left hemisphere lesions (Heilman et al. 1984; Heilman and Valenstein 1979; Bisiach and Luzzatti 1978). In addition, a prominent role of the right hemisphere has been also observed in perceptual processing of simple visual features (Corballis et al. 2002; Nicholls et al. 1999) and in spatial representation (Nicholls and Roberts 2002; Jewell and McCourt 2000; McCourt and Jewell 1999; McCourt and Olafson 1997; Mattingley et al. 1994).

More recent lines of research have linked visuo-spatial performance to oscillatory neurophysiological activity in the alpha range (7–13 Hz) (Brüers and Van Rullen 2018; Van Rullen 2016) and converge on the notion that alpha oscillatory parameters are related to different aspects of perceptual visual processing (Pfurtscheller et al. 1994). For instance, the individual alpha frequency of occipital oscillations represents a measure of temporal resolution of visual perception (Cecere et al. 2015; Samaha and Postle 2015; Klimesch et al. 2007; Valera et al. 1981), whereas alpha power (Romei et al. 2008a, b) and phase (Mathewson et al. 2009, 2012; Bush et al. 2009) reflect variations in cortical excitability and visual awareness. In addition, alpha power lateralization has been interpreted as a visuo-attentional mechanism (Capilla et al. 2014) facilitating stimulus processing (Sauseng et al. 2005; Vazquez Marrufo et al. 2001) and suppressing irrelevant distractors (Jensen and Mazaheri 2010; Klimesch et al. 2007).

However, alpha rhythm has also been reported to represent the dominant EEG pattern during eyes-closed resting condition in healthy awake individuals, with a prominent distribution over posterior regions of the scalp (Rosanova et al. 2009; Berger 1929). Interestingly, recent perspectives have proposed an association between alpha power at rest and the tonic and distributed synchronous activity of the underlying neurons (Sadaghiani and Kleinschmidt 2016; Klimesh et al. 2007), possibly indexing active suppression of neural predictions in the visual system (Sadaghiani and Kleinschmidt 2016) and, therefore, reflecting an active engagement of the neurons of the underlying neural population.

Although accumulating evidence indicates a robust functional link between alpha oscillations and the activity in the visual system, proof of asymmetries in alpha oscillatory patterns is still scarce. Indeed, asymmetries in the alpha range have been mainly investigated in frontoparietal networks and has been linked to psychiatric conditions (Ocklenburg et al. 2019; Stewart et al. 2011; Bruder et al. 2005, 2007; Metzeger et al. 2004) or hand preference (Ocklenburg et al 2019; Papousek and Shoulter 1999). However, recent findings on spatial orienting following directional cues have shown that, while in the left hemisphere alpha power decreases to facilitate visual processing in the contralateral field, alpha activity in the right hemisphere has a dual role in attention shifts. More precisely, alpha oscillations in the right hemisphere can both decrease, to enhance cued stimulus detection in the contralateral field, and increase to inhibit distractors in the contralateral field, when attention is cued to the ipsilateral field (Gallotto et al. 2020). On the other hand, a few studies also report imbalanced alpha activity at rest, with evidence of greater alpha power in the right hemisphere (Çiçek et al. 2003; Nalçaci et al. 1995; O’Boyle et al. 1991). Notably, recent evidence on patients with posterior brain lesions and hemianopia demonstrated that lesions of the posterior cortices result in a pathological resting eyes-closed alpha oscillatory pattern, with a slowdown of the individual alpha frequency peak (IAF) and a reduction of the amplitude in the lesioned hemisphere, which was more pronounced in hemianopics with right lesions, compared to hemianopics with left lesions (Pietrelli et al. 2019). This observation confirms that alpha oscillations at rest might reflect the functionality of the posterior cortices and that right posterior lesions have a more detrimental effect in altering the functioning of the visual system, in line with the hypothesis of a dominant role of the right hemisphere in visuo-spatial processing. However, the evidence showing that posterior lesions alter alpha oscillatory parameters (Pietrelli et al. 2019) raise the question whether the residual alpha recorded in hemianopic patients during eyes-closed resting state can retain some functionality and whether hemispheric asymmetries might be evident in this residual functioning.

A typical reactivity measure at rest is the decrease of alpha amplitude at the opening of the eyes (Ben-Simon et al. 2012; Barry et al. 2007), which is known as alpha desynchronization or alpha suppression (Berger 1929). This effect represents a basic physiological response at the opening of the eyes in normal conditions and is prominently observed over the posterior areas of the brain (Ben-Simon et al. 2012; Marx et al. 2003), but occurs all over the scalp without evident focal topographical changes (Barry and De Blasio 2017; Barry et al. 2007). In addition to alpha suppression, the opening of the eyes also induces changes in non-alpha low-frequency bands, which typically show a more focal distribution (Barry and De Blasio 2017; Barry et al. 2007). In particular, local desynchronization in the transition from the eyes-closed to the eyes-open condition has also been observed in the theta band and has been associated with low-level stimulus processing (Barry and De Blasio 2017; Gevins et al. 1997; Grillon and Buchsbaum 1986). In this perspective, the typical alpha desynchronization at the opening of the eyes represents a widespread cortical activation, enabling focal changes in non-alpha bands (e.g. in the theta band) to gather visual information (Barry and De Blasio 2017; Barry et al. 2007; Marx et al. 2003). The complexity of this global and local oscillatory changes at eyes-opening may thus reflect increased active engagement of visual system (Barry and De Blasio 2017) and this engagement has been linked to widespread cortical and subcortico-cortical interactions (Başar 1999; Klimesch 1999).

Although investigations on alpha reactivity on clinical populations are limited, alterations in alpha reactivity were found in dementia (van der Hiele et al. 2008) and schizophrenia (Colombo et al. 1989). However, little is known about how brain lesions impact the EEG reactivity caused by eyes-opening. Nevertheless, lesion studies on this topic could be especially relevant to advance our understanding on the role of specific cortical sites and the contribution of each hemisphere to the generation, distribution and functionality of the alpha rhythm on the scalp.

For this reason, the present study tested whether damage to posterior cortices results in disrupted or altered alpha desynchronization in the transition from the eyes-closed to the eyes-open resting state, investigating separately the effects of left and right hemispheric lesions. In addition, local changes in non-alpha bands (theta band) at the opening of the eyes were also investigated, since alteration in the widespread alpha suppression might also induce modifications in the typical patterns of changes in lower frequency bands. To this aim, a group of hemianopic patients with posterior left lesions, a group of hemianopics with posterior right lesions, a control group of patients with more anterior lesions and a control group of healthy participants were tested, recording EEG during rest, both during eyes-closed and eyes-open conditions. Both widespread cortical reactivity in the alpha range and local oscillatory changes in the theta range at the opening of the eyes were assessed, to investigate the effects of both left and right posterior lesions on the complex interaction between global and local processes reflecting task-independent activation of the visual system. Finally, visual performance in hemianopics was also tested, to investigate whether possible alterations in EEG reactivity at the opening of the eyes can relate to residual visual detection abilities.

## Methods

### Participants

Four groups of participants took part to the study: 13 patients with visual field defects due to lesions to the left posterior cortices (10 males, mean age = 53.8 years, SD = 15.89; mean time since lesion onset = 12.7 months, SD = 11.85), 13 patients with visual field defects due to lesions to the right posterior cortices (10 males, mean age = 58.9 years, SD = 16.47; mean time since lesion onset = 12.5 months, SD = 14.18), a control group of 14 patients without hemianopia with fronto-temporal lesions sparing the posterior cortices (6 males, mean age = 47.9 years, SD = 11.49; mean time since lesion onset = 25 months, SD = 21.35), and a control group of 14 age-matched healthy participants (7 males, mean age = 54.3 years, SD = 6.65). No differences between the groups were found in terms of age (*F*_3,50_ = 1.36; *p* = 0.212) or time since lesion onset (*F*_2,37_ = 2.58; *p* = 0.089; for clinical details, please see Table S1, Supplementary information).

**Fig. 1:**
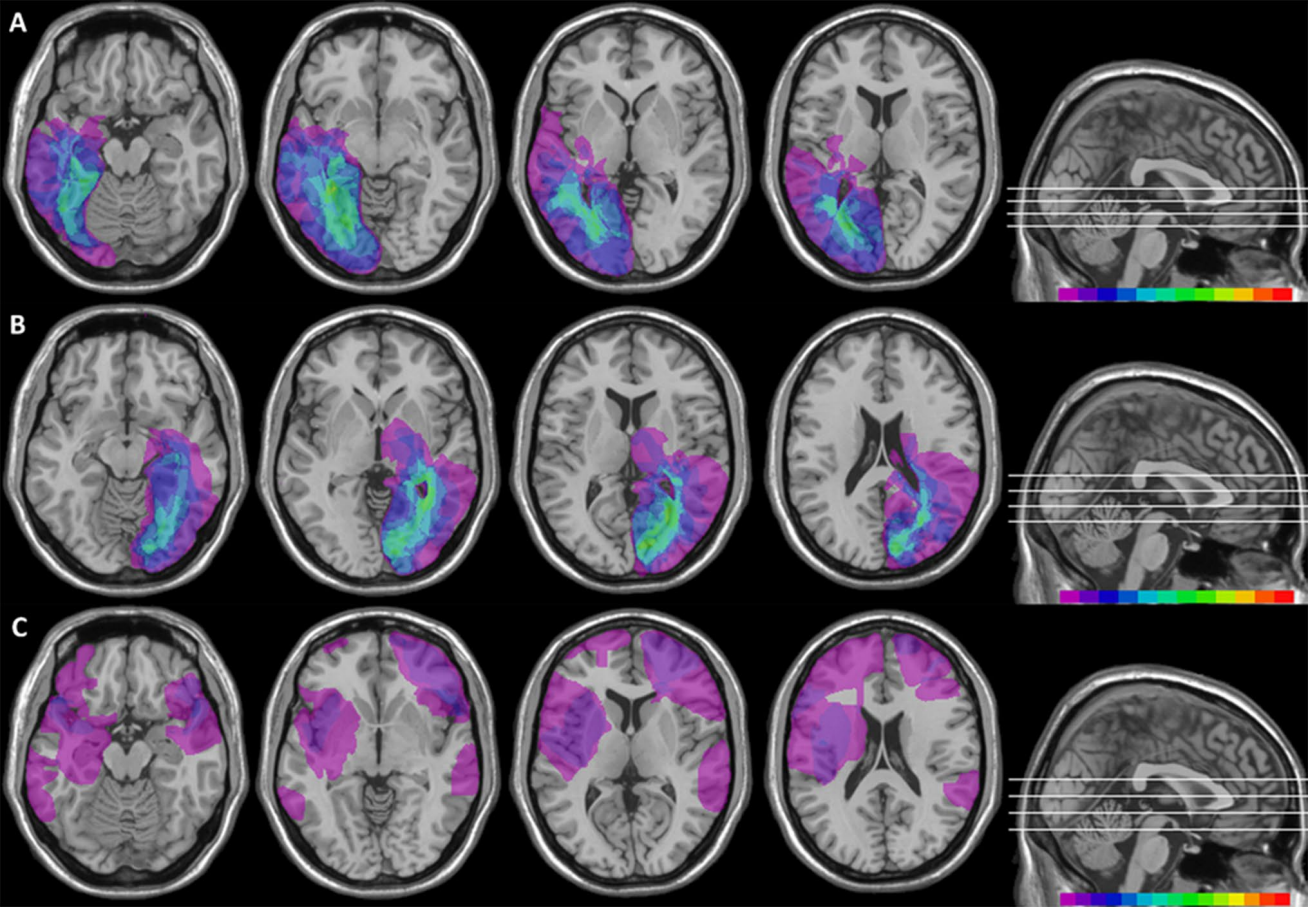
Location and overlap of brain lesions of patients. The image shows the lesions of the hemianopic patients with left posterior lesions **a**, hemianopic patients with right posterior lesions **b** and control patients with anterior brain lesions **c** projected onto four axial slices of the standard MNI brain. In each slice, the left hemisphere is on the left side. The levels of the axial slices are marked by white lines on the sagittal view of the brain. The color bar indicates the number of overlapping lesions

Mapping of brain lesions was performed using MRIcro. Lesions documented by the most recent clinical CT or MRI were traced onto the T1-weighted MRI template from the Montreal Neurological Institute provided with MRIcro software (Rorden et al. 2007; Rorden and Brett 2000), with the exception of HEMI7 and HEMI21 whose MRI scans were not available. Lesion volumes were computed for each patient to compare the extension of the lesions among the three patients’ groups. No significant differences (one-way ANOVA, *F*_2,35_ = 0.90; *p* = 0.414) among left-lesioned hemianopic patients, right-lesioned hemianopic patients and control patients were found (Fig. 1). Patients with posterior lesions were recruited based on reported visual field defects, the availability of a visual field perimetry and CT/MRI reports of the lesion. In patients with right lesions, the presence of neglect was screened using the Behavioral Inattention Test (Wilson et al. 1987), to ensure performance was in the normal range (conventional subscale: *M = *136.8; SD = 6.2. Behavioral subscale: *M = *75; SD = 5.4).

All patients showed normal or corrected-to-normal visual acuity. Patients were informed about the procedure and the purpose of the study and gave written informed consent. The study was designed and performed in accordance with the ethical principles of the Declaration of Helsinki and was approved by the Ethics Committee of the Regional Health Service Romagna (CEROM; n.2300).

### EEG during resting-state

Participants comfortably seated at rest in a sound-proof room in front of a 24′′ LCD monitor (refresh rate 60 Hz) at a viewing distance of 57 cm. EEG signal was recorded in five sessions of one-minute for each of the two resting conditions: eyes-closed and eyes-open resting state. During the eyes-open resting state, participants were asked to fixate a white central fixation cross (0.5°) against a black background on the monitor. The two resting conditions were alternated among the one-minute session of recording. EEG data were acquired through a BrainAmp DC amplifier (BrainProducts GmbH, Germany) and Ag/AgCl electrodes (*F*ast’nEasy Cap, Easycap GmbH, Germany) from 59 scalp sites (Fp1, AF3, AF7, F1, F3, F7, FC1, FC3, FC5, FT7, C1, C3, C5, T7, CP1, CP3, CP5, TP7, P1, P3, P5, P7, PO3, PO7, O1, Fp2, AF4, AF8, F2, F4, F8, FC2, FC4, FC6, FT8, C2, C4, C6, T8, CP2, CP4, CP6, TP8, P2, P4, P6, P8, PO4, PO8, O2, FPz, AFz, Fz, FCz, Cz, CPz, Pz, POz, Oz) and the right mastoid. The left mastoid was used as online reference electrode, while the ground electrode was placed on the right cheek. Vertical and horizontal electrooculogram (EOG) components were recorded from above and below the left eye, and from the outer canthus of each eye. Data were recorded with a band-pass filter of 0.01–100 Hz and digitized at a sampling rate of 1000 Hz, while impendences were kept under 5 KΩ. Raw EEG signal was off-line pre-processed and analyzed with EEGLAB (4.1.2b; Delorme and Makeig 2004), using custom Matlab routines (R2017a; The Mathworks Inc., USA). Data from all electrodes were re-referenced to the average of all scalp electrodes (Lasaponara et al. 2018, 2019; Pietrelli et al. 2019; Pirondini et al. 2020) and filtered with a band-pass filter of 1–100 Hz. The first 10 s of each one-minute recording session were excluded from the analysis, to avoid any contamination of the signal related to the transition from eyes-closed to the eyes-open resting condition. Continuous signal was segmented in epochs of 2 s. Horizontal and vertical eye artifacts were visually identified and removed by means of Independent Component Analysis (ICA), after data dimensionality reduction to 32 components based on Principal Component Analysis (PCA). On the cleaned EEG signal, a Fast Fourier Transformation (*F*FT) was computed on the 2-s epochs, with a frequency resolution of 0.5 Hz. Then, the amplitude of alpha and theta oscillations was calculated as the average power (in dB) in each electrode between 7 and 13 Hz 4 and 6 Hz, respectively. To compare the lesioned and intact hemispheres across participants, electrodes were swapped cross-hemispherically for patients with lesions to the right hemisphere (i.e., the data were analyzed as if all patients were left-lesioned).

**Fig. 2:**
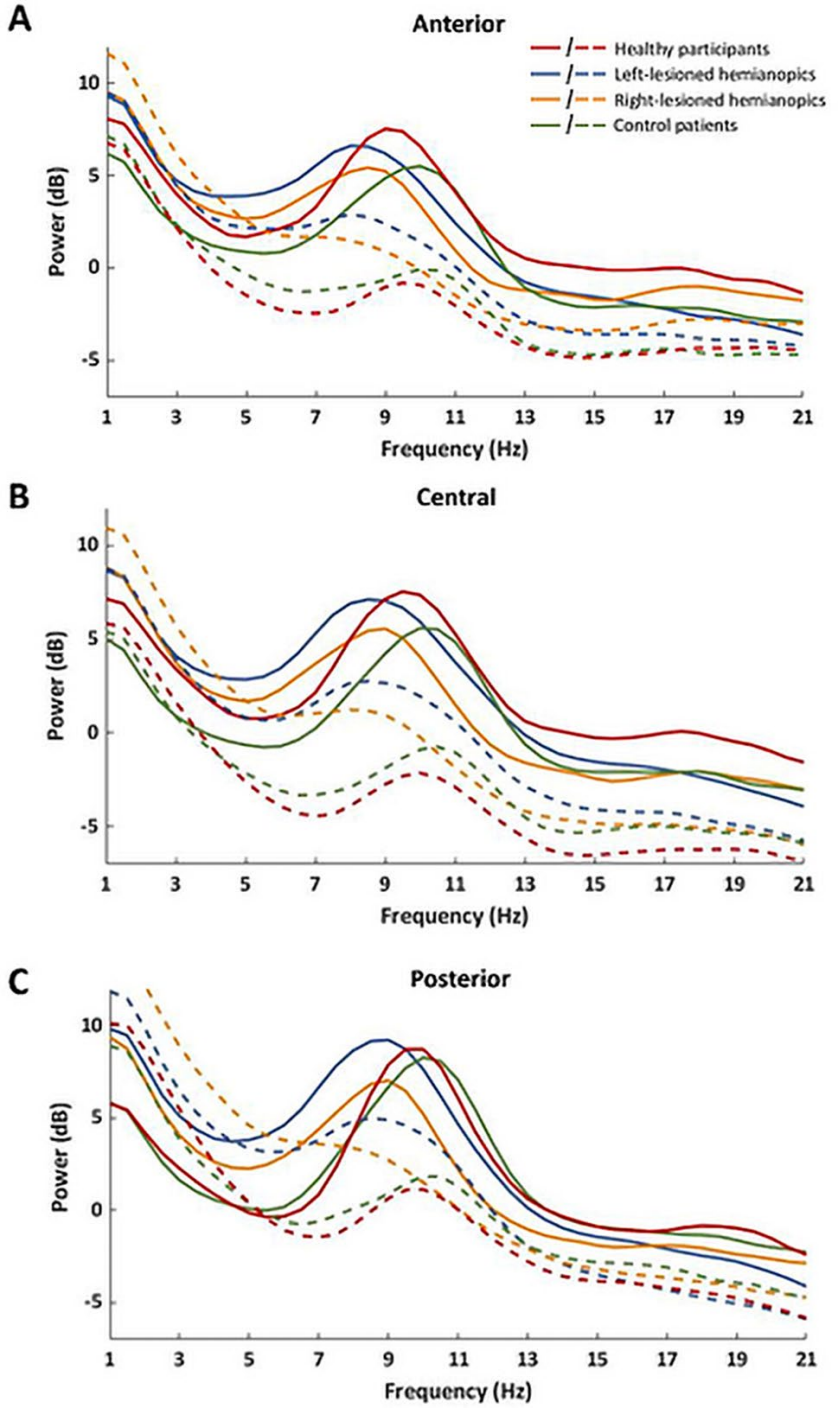
Spectrograms of the mean power across electrodes of the anterior **a**, central **b** and posterior **c** region of interest. Solid lines represent signal in the eyes-closed condition; dashed lines represent signal in the eyes-open condition

All the electrodes on the scalp were considered into the subsequent analyses, with the exception of the more anterior electrodes, to avoid contamination of the signal by the ocular artifacts, and electrodes on the sagittal midline, to provide a better segregation of the signal between the two hemispheres. The remaining electrodes were divided in six regions of interest (ROI), to perform statistical analysis on alpha and theta power on the entire scalp (Fig. 2). Six right (P4, P6, P8, PO4, PO8, O2) and left (P3, P5, P7, PO3, PO7, O1) parieto-occipital electrodes were grouped in two ROIs representing the posterior regions of the intact/right hemisphere and the lesioned/left hemisphere, respectively. Similarly, centro-parietal right (C2, C4, C6, CP2, CP4, CP6, P2) and left (C1, C3, C5, CP1, CP3, CP5, P1) electrodes and anterior right (AF4, F2, F4, FC2, FC4, FC6) and left (AF3, F1, F3, FC1, FC3, FC5) electrodes were grouped in four ROIs representing central and anterior regions of the intact/right and lesioned/left hemisphere, respectively.

To test whether posterior brain damage might affect modulation of alpha and theta power induced by eyes-opening, the oscillatory EEG power in both frequency bands was analyzed with separate ANOVAs with CONDITION (eyes-closed, eyes-open), HEMISPHERE (lesioned, intact) and ROI (posterior, central, anterior), as within subject factors and GROUP (healthy participants, hemianopic patients with left posterior lesions, hemianopic patients with right posterior lesions, control patients with anterior lesions) as between subjects factor. Post-hoc comparisons were performed with either Tukey or Tukey HSD, in case of unequal sample size.

### Computerized visual field test

In addition to the EEG recording, during the clinical examination, visual detection abilities in hemianopic patients were also tested, to investigate a possible link between visual performance and EEG reactivity at the opening of the eyes (Bolognini et al. 2005; Passamonti et al. 2009). Patients were presented with a stimulus array of 52° × 45° (horizontally and vertically, respectively), projected on the wall at a viewing distance of 120 cm. Targets consisted of white dots (1°), presented for 100 ms at different positions on a black background. A red fixation cross (0.5°) was presented on the center of the screen. The total number of targets presented was 96 (i.e. 48 targets for each hemifield). No target was presented in 31 trials (i.e., catch trials). Patients were asked to press a response button after the detection of the target. The task was performed in two different conditions: when patients were not allowed to move their eyes to compensate for the visual field loss and had to keep their gaze on a central fixation cross (fixed-eyes) and when patients were allowed to perform eye movements (eye movements). The experimenter monitored the patients’ gaze throughout the task. Visual detections and false alarms rates were measured. D prime (perceptual sensitivity) was calculated and used for subsequent correlational statistical analysis with EEG indices.

## Results

### Alpha power in the eyes-closed and in the eyes-open resting conditions

The overall ANOVA on alpha power revealed a significant main effect of CONDITION (*F*_1,50_ = 157.73; *p < *0.001; ηp^2^ = 0.76), with higher alpha power in eyes-closed condition (*M = *3.97 dB) compared to the eyes-open condition (*M = *− 0.53 dB; *p < *0.001), showing the presence of a significant desynchronization of the power of alpha at the opening of the eyes. In addition, a significant main effect of ROI (*F*_2,100_ = 61.09; *p < *0.001; η*p*^2^ = 0.55) was found, explained by higher power in posterior regions (*M = *2.81 dB), relative to central regions (*M = *1.06 dB; *p < *0.001) and anterior regions (*M = *1.30 dB; *p < *0.001). On the contrary, no significant main effect of GROUP (*F*_3,50_ = 0.86, *p* = 0.467; η*p*^2^ = 0.05) nor HEMISPHERE (*F*_1,50_ = 3.16; *p* = 0.081; η*p*^2^ = 0.06) was found.

**Fig. 3:**
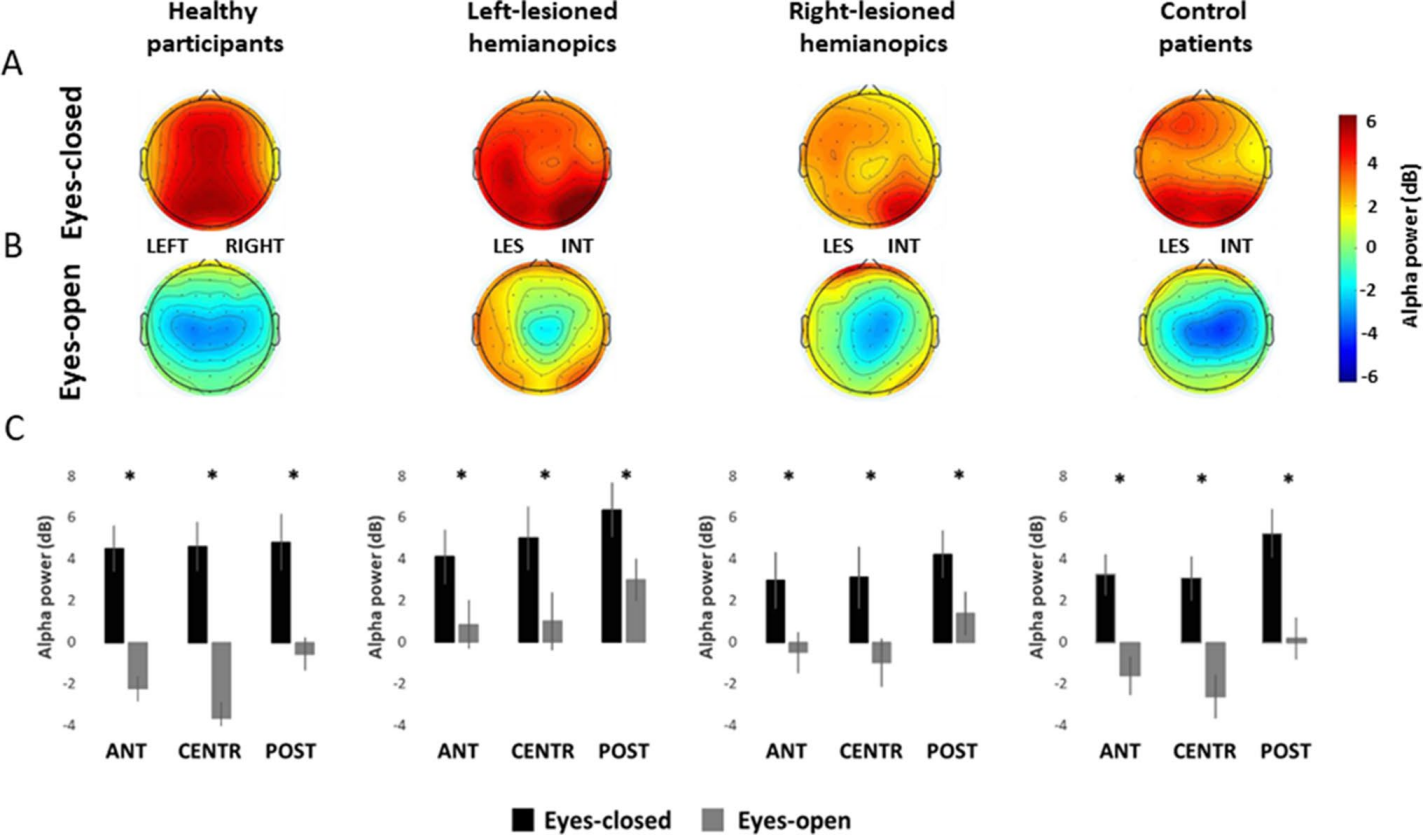
Scalp topographies represent the scalp distribution of the alpha power (dB) within each group in the frequency window 7–13 Hz, in the eyes-closed condition **a** and in the eyes-open condition **b**. For patients with lesions to the right hemisphere, electrodes were swapped cross-hemispherically, so that the lesioned hemisphere is represented on the left side. **c** Bar histograms show the mean alpha power (dB) in the eyes-closed and the eyes-open conditions, relative to anterior, central and posterior region of interest, within each group. Error bars represent standard error; asterisks are signaling the significant comparisons. *ANT* anterior region of interest, *CENTR* central region of interest, *POST* posterior region of interest, *LES* lesioned hemisphere, *INT* intact hemisphere

Significant CONDITION×GROUP (*F*_3,50_ = 6.12; *p* = 0.001; η*p*^2^ = 0.27), HEMISPHERE×GROUP (*F*_3,50_ = 2.83; *p* = 0.048; η*p*^2^ = 0.15) and CONDITION×HEMISPHERE×GROUP (*F*_3,50_ = 3.64; *p* = 0.019; η*p*^2^ = 0.18) interactions were also found. More importantly, the ANOVA revealed a significant CONDITION×ROI×GROUP (*F*_6,100_ = 4.22; *p < *0.001; η*p*^2^ = 0.20) interaction (Figs. 2 and 3).

To investigate the distribution of alpha desynchronization over the regions of the scalp within each group, this latter significant interaction was explored, performing separate ANOVAs on each group of participants, with CONDITION (eyes-closed, eyes-open) and ROI (posterior, central, anterior) as factors.

The ANOVA on the group of healthy participants revealed a significant main effect of CONDITION (*F*_1,13_ = 85.45; *p < *0.001; η*p*^2^ = 0.87), with higher alpha power in the eyes-closed condition (*M = *4.61 dB) compared to the eyes-open condition (*M = *− 2.1 dB; *p < *0.001), indicating a significant desynchronization all over the scalp at the opening of the eyes. Moreover, a significant main effect of ROI (*F*_2,26_ = 20.46; *p < *0.001; η*p*^2^ = 0.61) was found, with higher alpha power over posterior regions (*M = *2.11 dB), relative to central regions (*M = *0.48 dB; *p < *0.001) and anterior regions (*M = *1.13 dB; *p < *0.001) and higher alpha power in anterior regions, compared to central regions (*p* = 0.043). Also, a significant CONDITION×ROI (*F*_2,26_ = 47.81; *p < *0.001; η*p*^2^ = 0.77) interaction was found. Post-hoc comparisons showed significantly higher alpha power in the eyes-closed condition compared to the eyes-open condition in posterior regions (eyes-closed *M = *4.78 dB; eyes-open *M = *− 0.56 dB, *p < *0.001), central regions (eyes-closed *M = *4.57 dB; eyes-open *M = *-3.61 dB, *p < *0.001) and anterior regions (eyes-closed *M = *4.47 dB; eyes-open *M = *− 2.20 dB; *p < *0.001; Fig. 3). In addition, in the eyes-open condition, alpha power in posterior regions was significantly higher than in parietal regions (*p < *0.001) and anterior regions (*p < *0.001; Fig. 3).

The ANOVA on hemianopic patients with left lesions revealed a significant main effect of CONDITION (*F*_1,12_ = 23.14; *p < *0.001; η*p*^2^ = 0.66) with higher alpha power in the eyes-closed condition (*M = *4.69 dB) compared to the eyes-open condition (*M = *1.46 dB; *p < *0.001), again indicating a significant desynchronization all over the scalp at the opening of the eyes. In addition, a significant main effect of ROI (*F*_2,24_ = 13.05; *p < *0.001; ηp^2^ = 0.52) was found, with higher alpha power in posterior regions (*M = *4.26 dB), relative to central regions (*M = *2.73 dB; *p* = 0.003) and anterior regions (*M = *2.25 dB; *p < *0.001). On the contrary, the CONDITION×ROI (*F*_2,24_ = 1.89; *p* = 0.180; η*p*^2^ = 0.13) interaction was not significant (Fig. 3).

Similarly to the ANOVAs on healthy participants and hemianopic patients with left lesions, the ANOVA on the group of hemianopics with right lesions showed again a significant main effect of CONDITION (*F*_1,12_ = 30.12; *p < *0.001; η*p*^2^ = 0.71), with higher alpha power in the eyes-closed condition (*M = *2.92 dB) compared to the eyes-open condition (*M = *− 0.20 dB; *p < *0.001) and a significant main effect of ROI (*F*_2,24_ = 11.64; *p < *0.001; ηp^2^ = 0,49), explained by higher alpha power in posterior regions (*M = *2.38 dB), relative to central regions (*M = *0.91 dB; *p < *0.001) and anterior regions (*M = *1.06 dB; *p < *0.001). The CONDITION×ROI interaction (*F*_2,24_ = 3.05; *p* = 0.066; η*p*^2^ = 0.20) was not significant (Fig. 3).

Finally, also the ANOVA on controls patient with anterior lesions showed a significant main effect of CONDITION (*F*_1,13_ = 34.36; *p < *0.001; η*p*^2^ = 0.72), with higher alpha power in the eyes-closed condition (*M = *3.64 dB), compared to the eyes-open condition (*M = *− 1.25 dB; *p < *0.001) and a significant main effect of ROI (*F*_2,26_ = 23.00; *p < *0.001; η*p*^2^ = 0.64), revealing higher alpha power in posterior regions (*M = *2.56 dB), compared to central (*M = *0.24 dB; *p < *0.001) and anterior regions (*M = *0.79 dB; *p < *0.001). No significant CONDITION×ROI (*F*_2,26_ = 1.80; *p* = 0.185; η*p*^2^ = 0.12) interaction was found (Fig. 3).

### Alpha power reactivity

The results of the statistical analysis on the alpha power in the eyes-closed and in the eyes-open conditions suggest the presence of a significant alpha power desynchronization induced by the opening of the eyes all over the scalp, both in healthy participants and in hemianopic and control patients. However, to compare the magnitude of the alpha desynchronization at the opening of the eyes between groups, an index of alpha reactivity was further calculated. The alpha reactivity index was computed by subtracting the mean power in the eyes-open condition to the mean power in the eyes-closed condition (alpha reactivity = mean alpha power eyes-closed minus mean alpha power eyes-open) in each ROI separately, for each group of participants.

One-way ANOVAs were performed for the posterior, the central and the anterior ROIs, with GROUP (healthy participants, hemianopic patients with left posterior lesions, hemianopic patients with right posterior lesions, control patients with anterior lesions) as between-subject factor.

The ANOVA on the posterior ROI revealed a significant main effect of GROUP (*F*_3,50_ = 4.31; *p* = 0.009; η*p*^2^ = 0.21), pointing to a reduced alpha reactivity in right-lesioned hemianopic patients (*M = *2.22 dB), compared to healthy participants (*M = *5.34 dB; *p* = 0.018). In contrast, alpha reactivity in left-lesioned hemianopic patients (*M = *3.08 dB; *p* = 0.096) and control patients (*M = *4.75 dB; *p* = 0.917) was not significantly different relative to healthy participants. No other significant comparison was found (all ps > 0.081).

The ANOVA on the central ROI showed again a significant main effect of GROUP (*F*_3,50_ = 4.31; *p* = 0.009; η*p*^2^ = 0.20). In this region reduced alpha reactivity was found both in left-lesioned hemianopic patients (*M = *3.64 dB; *p* = 0.002) and in right-lesioned hemianopic patients (*M = *3.47 dB; *p* = 0.001), compared to healthy participants (*M = *8.17 dB). No significant difference in alpha reactivity was found between control patients (*M = *5.34 dB; *p* = 0.073) and healthy controls. No other comparison was significant (all ps > 0.394).

Finally, also the ANOVA on the anterior ROI showed a significant main effect of GROUP (*F*_3,50_ = 5.75; *p* = 0.002; η*p*^2^ = 0.26). Similarly to the results on the central ROI a reduced alpha reactivity was found only in left-lesioned hemianopic patients (*M = *2.96 dB; *p* = 0.006) and in right-lesioned hemianopic patients (*M = *2.94 dB; *p* = 0.005), compared to healthy participants (*M = *6.67 dB). No significant difference in alpha reactivity was found between control patients (*M = *4.56 dB; *p* = 0.183) and healthy controls. No other significant comparison was found (all ps > 0.428).

**Fig. 4:**
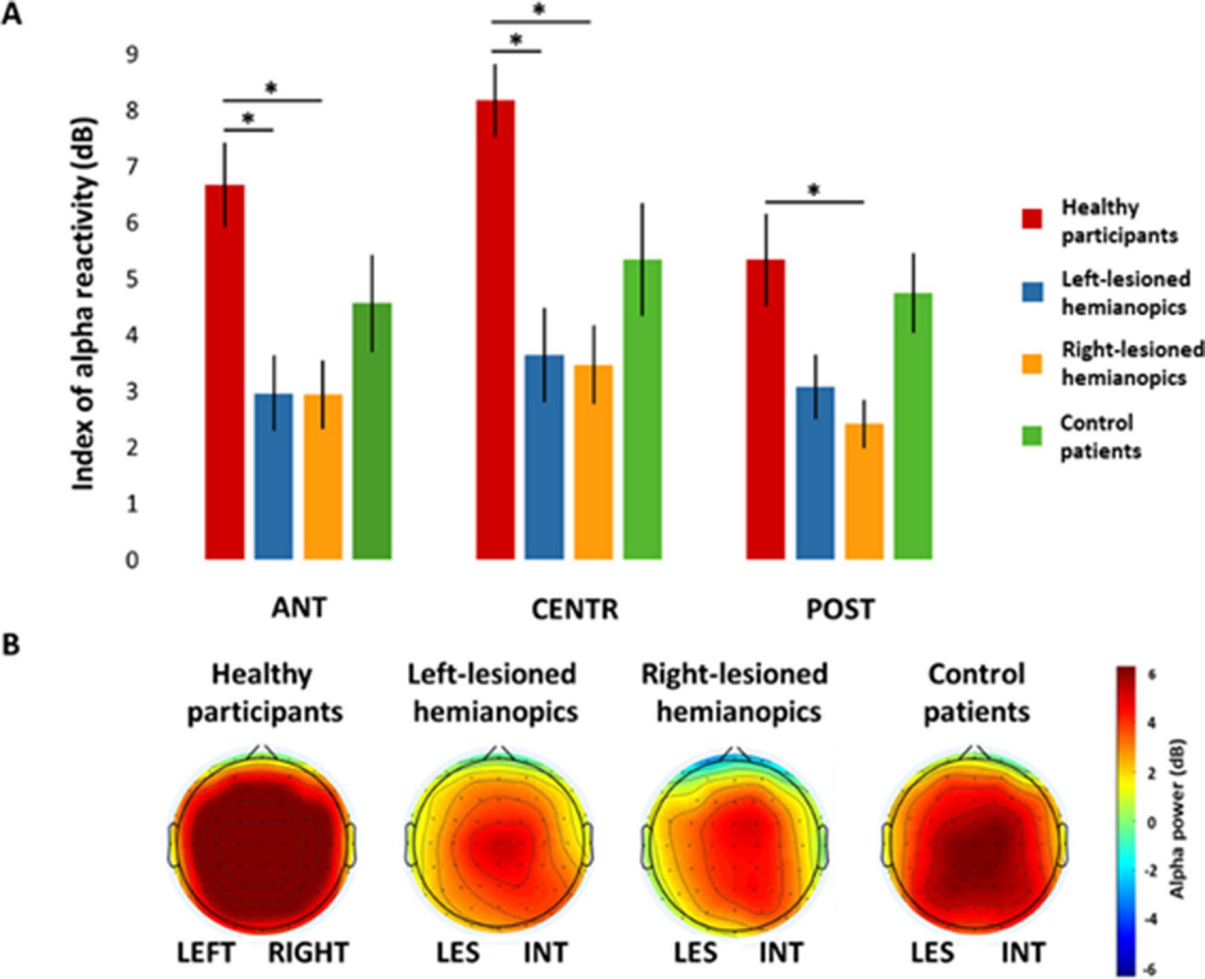
**a** Bar histograms show the index of alpha reactivity (dB) within each group, relative to the anterior, central and posterior region of interest and represent the comparisons between each group, separately for each region of interest. Error bars represent standard error; asterisks are signaling the significant comparisons. **b** Scalp topographies represent the scalp distribution of the magnitude of alpha power reactivity (dB; alpha reactivity = mean alpha power eyes-closed minus mean alpha power eyes-open) averaged across participants in the frequency window 7–13 Hz, in each group. For patients with lesions to the right hemisphere, electrodes were swapped cross-hemispherically, so that the lesioned hemisphere is represented on the left side. *ANT* anterior region of interest, *CENTR* central region of interest, *POST* posterior region of interest, *LES* lesioned hemisphere, *INT* intact hemisphere

Overall, these results suggest a reduced alpha reactivity only in hemianopic patients, with right-lesioned hemianopics showing a more global and widespread reactivity reduction, compared to left-lesioned hemianopics (Fig. 4).

### Theta power in the eyes-closed and in the eyes-open resting conditions

**Fig. 5:**
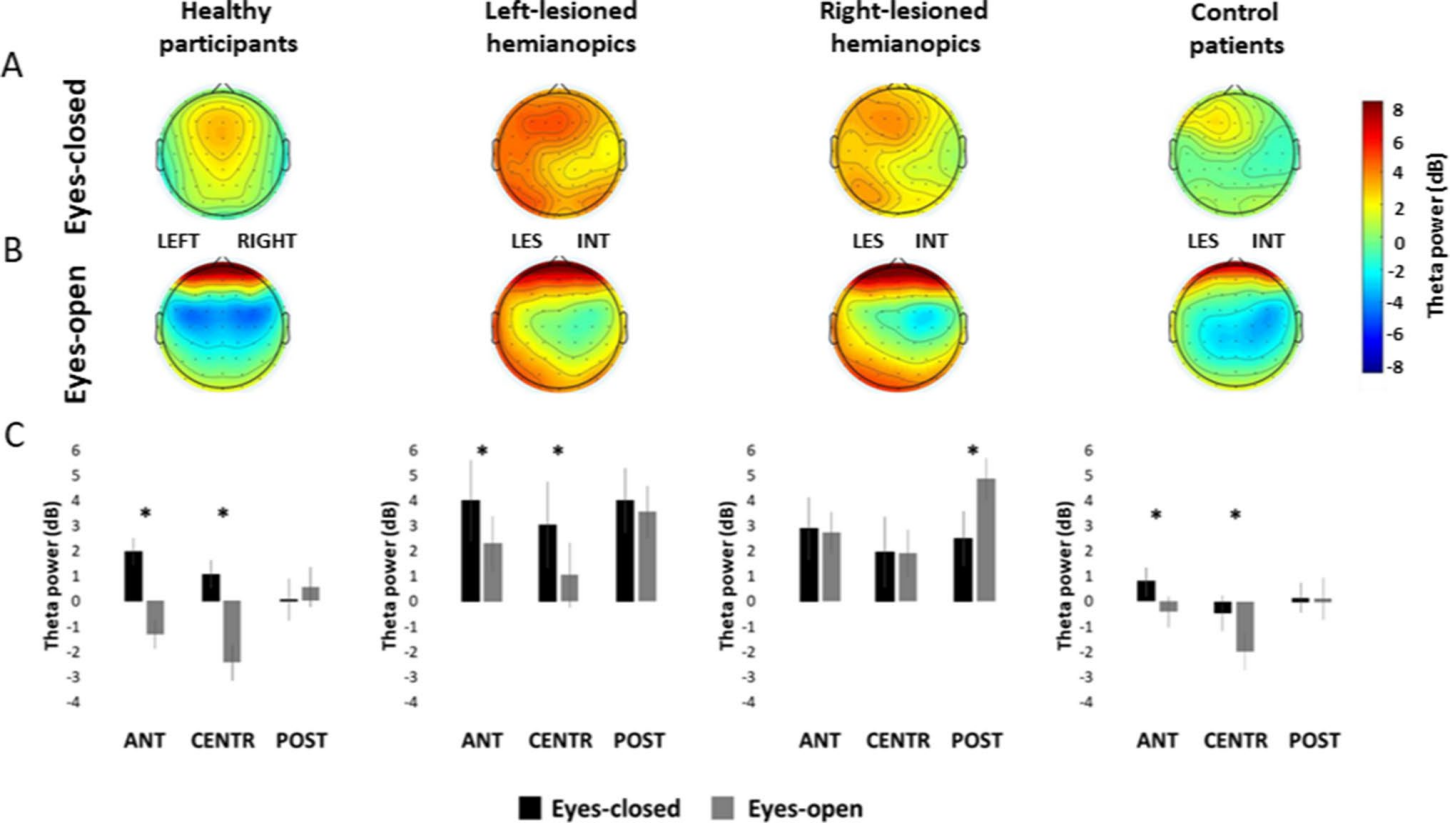
Scalp topographies represent the scalp distribution of the theta power (dB) within each group in the frequency window 4–6 Hz, in the eyes-closed condition **a** and in the eyes-open condition **b**. For patients with lesions to the right hemisphere, electrodes were swapped cross-hemispherically, so that the lesioned hemisphere is represented on the left side. **c** Bar histograms show the mean theta power (dB) in the eyes-closed and the eyes-open conditions, relative to anterior, central and posterior region of interest, within each group. Error bars represent standard error; asterisks are signaling the significant comparisons. *ANT* anterior region of interest, *CENTR* central region of interest, *POST* posterior region of interest, *LES* lesioned hemisphere, *INT* intact hemisphere

The overall ANOVA on theta power revealed a significant main effect of GROUP (*F*_3,50_ = 4.48; *p* = 0.007; η*p*^2^ = 0.21), with hemianopic patients with left lesions showing higher theta power (*M = *3.00 dB) relative to the control group of patients with anterior lesions (*M = *− 0.33; *p* = 0.041), while no other between-groups difference was evident (all ps > 0.075). Moreover, a significant main effect of CONDITION (*F*_1,50_ = 4.33; *p* = 0.042; η*p*^2^ = 0.08) was found, explained by higher theta power in the eyes-closed condition (*M = *1.80 dB) compared to the eyes-open condition (*M = *0.82 dB; *p* = 0.038), indicating a significant desynchronization of the power of theta at the opening of the eyes. A significant main effect of HEMISPHERE (*F*_1,50_ = 40.89; *p* =  < 0.001; η*p*^2^ = 0.45), with higher theta power in the lesioned/left hemisphere (*M = *1.72 dB) compared to the intact/right hemisphere (*M = *0. 89 dB; *p < *0.001) was also found. In addition, and a significant main effect of ROI (*F*_2,100_ = 43.96; *p* = 0.007; η*p*^2^ = 0.47) was found, revealing lower theta power in central regions (*M = *0.41 dB), compared to posterior regions (*M = *1.94 dB; *p < *0.001) and anterior regions (*M = *1.57 dB; *p < *0.001). Significant HEMISPHERE×GROUP (*F*_3,50_ = 4.59; *p* = 0.006; η*p*^2^ = 0.22) and HEMISPHERE×ROI×GROUP (*F*_6,100_ = 2.51; *p* = 0.026; η*p*^2^ = 0.13) interactions were also found. More importantly, the ANOVA revealed a significant CONDITION×ROI×GROUP (*F*_6,10_ = 3.0; *p* = 0.008; η*p*^2^ = 0.15, Fig. 5) interaction. Similarly, to the analyses on alpha power, this interaction was further explored, performing separate ANOVAs on each group of participants, with CONDITION (eyes-closed, eyes-open) and ROI (posterior, central, anterior) as factors, to investigate the distribution of theta desynchronization over the regions of the scalp within each group.

The ANOVA on the group of healthy participants showed a significant main effect of CONDITION (*F*_1,13_ = 6.05.; *p* = 0.028; η*p*^2^ = 0.32) with higher theta power in the eyes-closed condition (*M = *1.03 dB) compared to the eyes-open condition (*M = *− 1.07 dB; *p* = 0.028). In addition a significant main effect of ROI (*F*_2,26_ = 14.09; *p < *0.001; η*p*^2^ = 0.53) was found, with significantly lower theta power in central regions (*M = *− 0.70 dB) relative to posterior (*M = *0.31 dB; *p < *0.001) and anterior regions (*M = *0.32 dB; *p < *0.001). Finally, a significant CONDITION×ROI (*F*_2,26_ = 66.06; *p < *0.001; ηp^2^ = 0.84) interaction was found. Post-hoc comparisons revealed significantly higher theta power in the eyes-closed condition compared to the eyes-open condition in the anterior regions (eyes-closed *M = *1.97; eyes-open *M = *− 1.32 dB; *p < *0.001 dB) and the central regions (eyes-closed *M = *1.06 dB; eyes-open *M = *− 2.45 dB; *p < *0.001), but no significant difference between the two conditions was found in posterior regions (eyes-closed *M = *0.06 dB; eyes-open *M = *0.56; *p* = 0.498), indicating the presence of theta desynchronization only in anterior and central regions (Fig. 5).

The ANOVA on hemianopic patients with left lesions did not show a significant main effect of CONDITION (*F*_1,12_ = 2.27; *p* = 0.158; η*p*^2^ = 0.16), but a significant main effect of ROI (*F*_2,24_ = 2.36; *p < *0.001; η*p*^2^ = 0.51) with lower theta power in central regions (*M = *2.06 dB) compared to posterior regions (*M = *3.80 dB; *p < *0.001) and anterior regions (*M = *3.15; *p* = 0.013). In addition, the CONDITION×ROI (*F*_2,22_ = 4.66; *p* = 0.020; ηp^2^ = 0.29) interaction was also significant. Similarly to healthy participants, the subsequent post-hoc comparisons revealed significantly higher theta power in the eyes-closed condition compared to the eyes-open condition in the anterior regions (eyes-closed *M = *4.01 dB; eyes-open *M = *2.29 dB; *p* = 0.001) and in the central regions (eyes-closed *M = *3.05 dB; eyes-open *M = *1.06 dB; *p < *0.001), but not in posterior regions (eyes-closed *M = *4.02 dB; eyes-open *M = *3.57 dB; *p* = 0.822; Fig. 5).

The ANOVA on the group of hemianopic patients with right lesions did not show a significant main effect of CONDITION (*F*_1,12_ = 0.59; *p* = 0.456; ηp^2^ = 0.045), but a significant main effect of ROI (*F*_2,24_ = 9.11; *p* = 0.001; η*p*^2^ = 0.43) explained by higher theta power in posterior regions (*M = *3.70 dB) relative to central regions (*M = *1.95 dB; *p < *0.001), while no other significant difference among the regions was found (all ps > 0.102). In addition, the CONDITION×ROI (*F*_2,24_ = 9.30; *p* = 0.001; η*p*^2^ = 0.43) interaction was also significant. However, in contrast to the results on healthy participants and left-lesioned hemianopics, post-hoc comparisons did not reveal any significant difference in theta power between the eyes-closed and the eyes-open conditions in the anterior regions (eyes-closed *M = *2.89 dB; eyes-open *M = *2.74 dB; *p* = 0.999) and the central regions (eyes-closed *M = *1.97 dB; eyes-open *M = *1.93 dB; *p* = 1.00; Fig. 5). Moreover, a significant lower theta power in the eyes-closed condition (*M = *2.51 dB) compared to the eyes-open condition (*M = *4.88 dB; *p < *0.001) was found in posterior regions, indicating the presence of a significant theta synchronization at the opening of the eyes (Fig. 5).

Finally, in the control group of patients with anterior lesions, no significant main effect of CONDITION (*F*_1,13_ = 1.26; *p* = 0.28; η*p*^2^ = 0.09) was found. In contrast, a significant main effect of ROI (*F*_2,6_ = 13.77; *p < *0.001; η*p*^2^ = 0.52) was evident, with significantly lower theta power in central regions (*M = *-1.43 dB) compared to posterior (*M = *0.20 dB; *p < *0.001) and anterior regions (*M = *0.22 dB; *p < *0.001). The CONDITION×ROI (*F*_2,26_ = 5.43, *p* = 0.011; η*p*^2^ = 0.29) interaction was also significant. Again similarly to healthy participants and left-lesioned hemianopics, post-hoc comparisons revealed a significant higher theta power in the eyes-closed condition relative to the eyes-open condition in the anterior regions (eyes-closed *M = *− 0.95 dB; eyes-open *M = *− 0.50 dB; *p* = 0.013) and the central regions (eyes-closed *M = *− 0.54 dB; eyes-open *M = *− 2.32 dB; *p* = 0.002), but not in posterior regions (eyes-closed *M = *0.22 dB; eyes-open *M = *0.18 dB; *p* = 0.999; Fig. 5).

### Theta power reactivity

Overall, the results of the statistical analysis on the theta power in the eyes-closed and in the eyes-open conditions suggest differences between groups in theta power changes induced by the opening of the eyes in the three ROIs examined. More specifically, no changes between the eyes-closed and the eyes-open conditions were found in the posterior regions in all groups, with the exception of right-lesioned hemianopics, who showed an atypical increase in theta power at the opening of the eyes, compared to the eyes-closed condition. Differently, in the central and the anterior regions, all groups showed a significant desynchronization at the opening of the eyes, again with the exception of right-lesioned hemianopics, who did not show any significant change between the eyes-closed and the eyes-open conditions. An index of theta reactivity at the opening of the eyes was calculated (theta reactivity = mean theta power eyes-closed minus mean theta power eyes-open), to compare the magnitude of theta desynchronization at the opening of the eyes in the central and anterior regions among the groups of participants showing desynchronization (i.e., healthy participants, left-lesioned hemianopics and control patients with anterior lesions). Right-lesioned hemianopics were not included in this comparison, since they did not exhibit a significant desynchronization. Two separate one-way ANOVAs were performed for the central and the anterior ROIs, having GROUP (healthy participants, hemianopic patients with left posterior lesions, control patients with anterior lesions) as between-subjects factor.

Both the ANOVAs on the central ROI (*F*_2,38_ = 0.83; *p* = 0.44; *ηp*^*2*^ = 0.004) and the anterior ROI (*F*_2,38_ = 1.19; *p* = 0.31; η*p*^2^ = 0.006) did not show a significant main effect of GROUP. This suggests that the overall pattern of theta desynchronization was similar between left-lesioned hemianopics, patients with anterior lesions and healthy participants. On the contrary, right-lesioned hemianopic patients revealed an atypical pattern of theta changes at the opening of the eyes, with no desynchronization in the central and anterior regions and the presence of theta synchronization in the posterior regions.

### Hemianopic patients’ visual performance and reactivity in the alpha and theta band

Finally, we tested whether altered alpha and theta reactivity over the posterior, central and anterior ROIs can relate to behavioral performance in visual detection tests in hemianopic patients with both left and right lesions. To this aim, the relationship between hemianopic patients' perceptual sensitivity in their blind field at the computerized visual field test in the fixed-eyes condition and their indices of alpha and theta reactivity was explored separately for each ROI. Simple correlations were performed and, to account for multiple comparisons, *p* values were adjusted with Holm–Bonferroni corrections. Adjusted p values (adj. ps) are reported. No significant correlation between the mean D prime in the blind field and the indices of both alpha and theta reactivity was found in the three ROIs examined (all adj. ps > 0.171), suggesting that the residual alpha and theta reactivity in hemianopic patients is not associated with the sparing of their visual field. In addition, the relationship between hemianopic patients’ perceptual sensitivity in their blind field at the computerized visual field test in the eye-movements condition and their indices of alpha and theta reactivity was explored separately for each ROI. Again, simple correlations were performed, and, to account for multiple comparisons, p values were adjusted with Holm–Bonferroni corrections. Adjusted p values (adj. ps) are reported. No significant correlation between the mean D prime in the blind field and the indices of both alpha and theta reactivity was found in the three ROIs examined (all adj ps > 0.366), indicating no relationship between alpha and theta reactivity and patients’ ability to compensate for the field loss with eye movements.

## Discussion

The present EEG study compared eyes-closed and eyes-open resting conditions in posterior-lesioned patients with visual field defects and age-matched control anterior-lesioned patients and healthy participants. The results showed that all groups presented a significant desynchronization of alpha power at the opening of the eyes, across all scalp regions. Specifically, decreased alpha power during the eyes-open condition compared to the eyes-closed condition was found in posterior, central and anterior sites, in both the left and the right hemispheres. Nevertheless, alpha reactivity induced by eyes-opening was reduced in both left- and right-lesioned hemianopic patients. This may indicate that hemianopics are characterized by altered task-independent activation of the visual system. More precisely, left-lesioned hemianopic patients exhibited a reduced alpha reactivity in the anterior and the central scalp regions; whereas, right-lesioned hemianopics showed an overall reduction of alpha reactivity all over the scalp, i.e. in the anterior, central and posterior ROIs, suggesting a more pronounced and extended dysfunction after lesions to the right hemisphere.

The altered alpha reactivity in hemianopics is in line with previous evidence, demonstrating that left and right posterior brain lesions selectively impair alpha oscillatory parameters during eyes-closed resting state, resulting in a slowdown of IAF and an interhemispheric power imbalance, in favor of the intact hemisphere (Pietrelli et al. 2019). Importantly, the present results show that, regardless the presence of alterations to the baseline alpha oscillatory activity due to posterior lesions, hemianopic patients retain a residual reactivity in the alpha range to the opening of the eyes, which is evident, but reduced, after damage to the posterior cortices. This residual reactivity in the alpha band seems also in agreement with previous reports showing that hemianopic patients can retain stimulus-related alpha changes, induced by the presentation of stimuli in the blind field (Grasso et al. 2020; Sanchez-Lopez et al. 2019).

Converging evidence report that eyes-closed and eyes-open conditions correspond to distinct neurophysiological states and functional connectivity patterns (Xu et al. 2014; Jao et al. 2012). More precisely, eyes-closed resting state has been linked to a state of greater network integration, with reduced modularity and increased global efficiency (Xu et al. 2014; Bianciardi et al. 2009). In contrast, eyes-open resting state has been associated with greater modularity, which is thought to facilitate increased local efficiency, subserving task-dependent processing (Allen et al. 2018; Xu et al. 2014). In this perspective, the alpha desynchronization in the transition from the eyes-closed to the eyes-open condition might represent a widespread cortical activation, supporting the focal decreases in non-alpha bands, related to local processing which gathers visual information (Barry and De Blasio 2017; Barry et al. 2007; Marx et al. 2003). Thus, the present findings indicate that focal unilateral lesions to posterior cortices can induce global and widespread alterations in alpha cortical reactivity, in line with the notion of a central role of low-level visual cortices in coordinating and propagating alpha oscillations in the visual system (Hindriks et al. 2015).

Notably, right hemispheric posterior lesions result in a more severe reactivity reduction, distributed all over the scalp, while in left-lesioned patients, posterior cortices retain a normal alpha reactivity. This observation is in agreement with previous findings on hemianopics showing that posterior right lesions had more detrimental consequences on alpha oscillatory impairments, with stronger IAF reduction and interhemispheric power imbalance, relative to posterior left lesions (Pietrelli et al. 2019). This seems to suggest a specialization of the right hemisphere in generating and distributing alpha oscillatory patterns. In agreement, evidence has shown that the right hemisphere is capable of modulating alpha oscillations to both facilitate detection of visual stimuli and suppress visual irrelevant information (Gallotto et al. 2020), suggesting a more specialized role of this hemisphere in allocating visuo-spatial attentional resources and tuning visual perceptual abilities through alpha oscillatory patterns.

In addition, in the present study, patients with right lesions also showed a peculiar pattern of reactivity at the opening of the eyes in the theta frequency range. More precisely, while healthy participants, control patients and also hemianopics with left lesions demonstrated a typical desynchronization in the theta range over centro-anterior regions at the opening of the eyes (Barry and De Blasio 2017; Barry et al. 2007), hemianopics with right lesions revealed no significant change over central and anterior regions of the scalp and an atypical increase of theta power over posterior regions, in the transition from eyes-closed to eyes-open resting state. Focal alterations in the theta range after brain damage has been consistently reported in eyes-closed resting state, regardless the site of the lesion. Specifically, increased theta power in perilesional areas has been described in patients with stroke (Chu et al. 2015; Laaksonen et al. 2013; Dubovik et al. 2012; Tecchio et al. 2005; Butz et al. 2004), likely reflecting reorganization of the lesioned cortices (Rabiller et al. 2015; Carmichael and Chesselet 2002). Previous reports comparing hemianopics and control patients with anterior lesions also showed that post-lesional theta power increase at eyes-closed rest is evident after lesions both to posterior and anterior cortices (Pietrelli et al. 2019). However, the current findings show that theta reactivity to the opening of the eyes seems selectively compromised only after posterior right lesions, which adds to the right-lesioned hemianopics’ reduced alpha reactivity.

The dysfunctional reactivity in the theta range observed in right-lesioned hemianopics might reflect the disruption of the typical focal oscillatory changes occurring at the opening of the eyes, which have been associated with stimulus processing and, hence, to low-level unstructured responses to visual stimuli during eyes-open resting state (Barry and De Blasio 2017; Gevins et al 1997; Grillon and Buchsbaum 1986). The combination of impairments in the alpha and the theta range observed in hemianopics with right lesions suggests the presence of a stronger impairment in functional reactivity to the opening of the eyes, compared to hemianopic patients with left lesions, involving both global and local processes. Indeed, right posterior lesions seem to primarily weaken the typical reduction of alpha power at the opening of the eyes, reflecting the widespread cortical activation, gating and controlling visual inputs at the opening of the eyes; then, as a consequence, right lesions also impair focal theta reduction, which is linked with modular processing and local cortical activations (Barry and De Blasio 2017; Gevins et al. 1997; Grillon and Buchsbaum 1986). This seems in line with the notion that alpha oscillations, propagating from posterior visual cortices to higher-order cortical sites (Hindriks et al. 2015), might play a special role in coordinating widespread oscillatory activity and orchestrating focal processing in non-alpha frequency bands, which might support visual processing at the opening of the eyes (Barry and De Blasio 2017).

In this perspective, the present findings suggest a possible role of the intact right hemisphere in compensating the disruption of alpha oscillatory reactivity due to left posterior lesions. Indeed, we can speculate that in left-lesioned hemianopics the right intact hemisphere might contribute to preserve alpha oscillatory activity in the posterior cortices, with a consequent spared normal reactivity in the theta range.

Although these results suggests that alterations in the reactivity patterns at the opening of the eyes are a dysfunctional feature of patients suffering posterior brain damage, it is notable that reactivity indices both in the alpha and in the theta band did not show any correlation with visual performance, both when hemianopic patients were required to fixate a central fixation point and when exploratory eye movements were allowed. This finding is in line with evidence showing that alpha desynchronization at the opening of the eyes occurs independently of external sensory input, for instance in blind individuals (Hüfner et al. 2009) and in condition of complete darkness (Ben-Simon et al. 2013; Boytsova and Danko 2010; Marx et al. 2003). This is consistent with the hypothesis that alpha reactivity at the opening of the eyes does not reflect visual processing per se, but represents a prerequisite for alpha visual-related modulation by external sensory stimulation (Ben-Simon et al. 2012; Klimesch et al 2007). In line with this perspective, a wide body of research converges on the notion that various aspects of visual performance are rather linked with different alpha oscillatory parameters (Brüers and Van Rullen 2018; Van Rullen 2016), with individual alpha frequency representing a measure of temporal resolution of visual perception (Cecere et al. 2015; Samaha and Postle 2015; Klimesch et al. 2007; Valera et al. 1981) and alpha power (Romei et al. 2008a, b) and phase (Mathewson et al. 2009, 2012; Bush et al. 2009) reflecting variations in cortical excitability and visual awareness. As a consequence, the present findings suggest that EEG reactivity indices should be interpreted as intrinsic electrophysiological biomarkers of the functional effects of posterior brain lesions.

Overall, the current results add to previous knowledge on hemispheric asymmetries in visuo-spatial abilities (Duecker et al. 2015; Heilman and Van Den Abell 1980; Kinsbourne 1977) and suggest a prominent role of the posterior cortices of the right hemisphere in organizing and distributing oscillatory alpha activity, to support the local functioning of the visual system at rest. This is in favor of a dominance of the right hemisphere in perceptual and visuo-spatial processing (Corballis et al. 2002; Nicholls et al. 2002; Jewell and McCourt 2000; Gitelman et al. 1999; McCourt and Jewell 1999; McCourt and Olafson 1997; Nobre et al. 1997; Mattingley et al. 1994; Heilman et al. 1984; Heilman and Valenstein 1979; Bisiach and Luzzatti 1978; Bueichekú et al. 2020) and emphasize the underlying role of complex oscillatory patterns.

## Supplementary Information

Below is the link to the electronic supplementary material.

Supplementary file1 (DOCX 19 KB)

## Data Availability

The datasets generated during and/or analyzed during the current study are available from the corresponding author on reasonable request.

## Code availability

Custom codes used for analyses are available from the corresponding author on reasonable request.
