# Supplementary material for: Hemispheric differences in altered reactivity of brain oscillations at rest after posterior lesions
Source: Brain Struct Funct. 2021 Apr 24;227(2):709–23. doi: 10.1007/s00429-021-02279-8 (PMC8844183; doi:10.1007/s00429-021-02279-8)
Supplement: Supplementary file 1 — Supplementary file1 (DOCX 19 KB) [file 429_2021_2279_MOESM1_ESM.docx]

|  |  |  |  |  |  |  |
| --- | --- | --- | --- | --- | --- | --- |
| **ID** | **Sex** | **Age** | **Onset** | **Lesion Site** | **Visual Field Defect** | **Aetiology** |
| HEMI1  HEMI2  HEMI3  HEMI4  HEMI5  HEMI6  HEMI7  HEMI8  HEMI9  HEMI10  HEMI11  HEMI12  HEMI13 | M  M  F  M  M  M  M  M  F  M  F  M  M | 69  45  57  50  81  51  41  45  29  58  32  69  73 | 5  7  28  7  9  5  2  41  26  6  4  8  17 | Left Occipital  Left Temporal  Left Fronto-Temporo-Insular  Left Temporo-Occipito-Parietal  Left Occipito-Temporal  Left Fronto-Temporo-Occipital  Left Occipital  Left Fronto-Parieto-Temporal  Left Temporal  Left Temporo-Occipital  Left Parieto-Occipital  Left Temporo-Occipital  Left Temporo-Occipital | Right hemianopia  Right hemianopia  Right hemianopia  Upper right quadrantopia  Right hemianopia  Right hemianopia  Lower right quadrantopia  Right hemianopia  Upper right hemianopia  Right hemianopia  Right hemianopia  Right hemianopia  Right hemianopia | Ischaemic  Hemorragic  AVM  Ischaemic  Ischaemic  Abscess  Ischaemic  Hemorragic  AVM  Ischaemic  Ischaemic  Hemorragic  Hemorragic |
| HEMI14  HEMI15  HEMI16  HEMI17  HEMI18  HEMI19  HEMI20  HEMI21  HEMI22  HEMI23  HEMI24  HEMI25  HEMI26 | M  F  F  M  M  M  M  F  M  M  M  M  M | 56  38  37  58  81  51  60  73  77  30  59  76  70 | 3  13  4  18  7  4  29  8  6  53  5  7  5 | Right Occipital  Right Parieto-Occipital  Right Occipito-Temporo-Parietal  Right Temporo-Occipital  Right Occipital  Right Occipital  Right Temporo-Occipital  Right Temporo-Occipital  Right Fronto-Parietal  Right Temporal  Right Temporo-Occipital  Right Occipital  Right Occipital | Left hemianopia  Left hemianopia  Left hemianopia  Left hemianopia  Left hemianopia  Left hemianopia  Left hemianopia  Left hemianopia  Left hemianopia  Left hemianopia  Left hemianopia  Left hemianopia  Left hemianopia | Ischaemic  Hemorragic  Tumor  Ischaemic  Hemorragic  Tumor  Ischaemic  Ischaemic  Hemorragic  Hemorragic  Ischaemic  Abscess  Ischaemic |
| CON1  CON2  CON3  CON4  CON5  CON6  CON7  CON8  CON9  CON10  CON11  CON12  CON13  CON14 | F  F  M  F  F  F  M  M  F  M  M  F  M  F | 48  44  28  45  46  57  42  62  42  34  51  50  75  47 | 38  40  11  39  12  5  59  7  19  7  3  71  26  13 | Left Fronto-Insular  Left Frontal  Left Fronto-Parietal  Left Frontal  Left Temporal  Right Fronto-Insular  Right Frontal  Left Temporo-Insular  Right Frontal  Left Frontal  Right Temporo-Insular  Right Temporo-Fronto-Polar  Right Temporo-Insular  Right Frontal | No hemianopia  No hemianopia  No hemianopia  No hemianopia  No hemianopia  No hemianopia  No hemianopia  No hemianopia  No hemianopia  No hemianopia  No hemianopia  No hemianopia  No hemianopia  No hemianopia | Ischaemic  Tumor  Tumor  Tumor  Hemorragic  AVM  Abscess  Abscess  Tumor  Tumor  Tumor  Traumatic  Tumor  Abscess |

**Table S1**. **Supplementary Information**

Summary of clinical data of all patients that took part to the study. Legend: M = Male; F = Female; AVM = Arteriovenus Malformation.
